# Estimating Walking Speed in the Wild

**DOI:** 10.3389/fspor.2020.583848

**Published:** 2020-11-25

**Authors:** Loubna Baroudi, Mark W. Newman, Elizabeth A. Jackson, Kira Barton, K. Alex Shorter, Stephen M. Cain

**Affiliations:** ^1^Department of Mechanical Engineering, University of Michigan, Ann Arbor, MI, United States; ^2^School of Information, University of Michigan, Ann Arbor, MI, United States; ^3^Division of Cardiovascular Medicine, Department of Internal Medicine, University of Alabama, Birmimgham, AL, United States

**Keywords:** gait analysis, wearable sensors, physical activity, gait speed, free-living

## Abstract

An individual's physical activity substantially impacts the potential for prevention and recovery from diverse health issues, including cardiovascular diseases. Precise quantification of a patient's level of day-to-day physical activity, which can be characterized by the type, intensity, and duration of movement, is crucial for clinicians. Walking is a primary and fundamental physical activity for most individuals. Walking speed has been shown to correlate with various heart pathologies and overall function. As such, it is often used as a metric to assess health performance. A range of clinical walking tests exist to evaluate gait and inform clinical decision-making. However, these assessments are often short, provide qualitative movement assessments, and are performed in a clinical setting that is not representative of the real-world. Technological advancements in wearable sensing and associated algorithms enable new opportunities to complement in-clinic evaluations of movement during free-living. However, the use of wearable devices to inform clinical decisions presents several challenges, including lack of subject compliance and limited sensor battery life. To bridge the gap between free-living and clinical environments, we propose an approach in which we utilize different wearable sensors at different temporal scales and resolutions. Here, we present a method to accurately estimate gait speed in the free-living environment from a low-power, lightweight accelerometer-based bio-logging tag secured on the thigh. We use high-resolution measurements of gait kinematics to build subject-specific data-driven models to accurately map stride frequencies extracted from the bio-logging system to stride speeds. The model-based estimates of stride speed were evaluated using a long outdoor walk and compared to stride parameters calculated from a foot-worn inertial measurement unit using the zero-velocity update algorithm. The proposed method presents an average concordance correlation coefficient of 0.80 for all subjects, and 97% of the error is within ±0.2*m*· *s*^−1^. The approach presented here provides promising results that can enable clinicians to complement their existing assessments of activity level and fitness with measurements of movement duration and intensity (walking speed) extracted at a week time scale and in the patients' free-living environment.

## 1. Introduction

In the United States and worldwide, cardiovascular diseases are the main cause of death in adults (Mensah and Brown, 2007). Efforts to combat and prevent these health issues have shown physical activity to be vital in maintaining, improving, and even recovering cardiovascular health (Englesbe et al., 2015). The ability to assess an individual's level of physical activity is a critical component of clinical evaluations. Questionnaires are often used to gather information about activity level during daily life. However, among the large variety of existing questionnaires, it is uncertain whether these survey instruments are capable of representing reliable and valid quantification of a patient's activity level (Sattler et al., 2018). With recent technological progress in wearable sensing technology, fitness trackers offer a promising complementary tool for clinical assessments. Activity trackers are devices, usually in the form of a wristband, that are used to monitor physical activity in the free-living environment by deriving metrics, such as estimated energy consumption, distance traveled, or step count. These activity budgets can be used by clinicians to prescribe tailored treatments, as well as to monitor adherence to these prescriptions (Ayabe et al., 2008; Walker et al., 2016; Cadmus-Bertram, 2017). Summary metrics from wearable sensors provide insight into the type and duration of physical activity. However, to provide information for clinical assessment of overall health, quantitative metrics from key activities, such as walking speed should be accessible.

Walking is a central activity of daily living; it is an easy, safe, and accessible aerobic exercise and does not require specialized skills or equipment. Given the cardiovascular benefits of walking, this low impact activity is often prescribed for patients as a way to meet their required amount of physical activity (Murtagh et al., 2010). Walking speed can be used as a metric to capture the intensity of walking and serve as an effective surrogate to evaluate fitness (Morris and Hardman, 1997; Afilalo et al., 2016). The correlation of walking speed with functional decline has also made it a widely used predictor for various health issues (Bohannon, 1997; Graham et al., 2008). In addition to the strong association with well-being, gait speed can be obtained in a clinical environment, is cost-effective, and easy to interpret (Fritz and Lusardi, 2009). Clinical walking tests typically estimate walking speed by timing the subject as they walk a prescribed distance or by measuring the distance a subject can walk in a prescribed amount of time. However, the available space and time constraints imposed in clinical settings can lead to variations in the duration, distance traveled, and instructions provided to the patient when measuring speed (Vaney et al., 1996; Dean et al., 2001). High day-to-day variabilities in the results of the same tests have also been observed (Prahm et al., 2014). In most cases, a manual stopwatch is used to measure time, which can lead to significant measurement error when conducting short tests. Consequently, the different measurement approaches typically used in clinical settings do not provide consistent estimates of walking speed. Further, few studies have compared speed measured in the clinic to measurements during natural walking in a free-living environment (Brodie et al., 2016, 2017). As there are fundamental contextual differences between supervised (e.g., laboratory or clinic) and unsupervised (e.g., free-living) environments (Warmerdam et al., 2020), it is unclear whether the values of walking speed extracted in a clinical setting are representative of the true walking behavior of an individual. To further enhance clinical understanding of real-world gait performance, metrics from free-living observations must be provided.

The analysis of walking gait in the real world has benefited greatly from technological advancements in wearable technologies. In particular, with the development of micro-electromechanical systems (MEMS), inertial measurement units (IMUs) have become compact and low-cost, which make them instruments of choice for walking gait analysis (Chen et al., 2016). High-resolution measurements are now possible outside laboratory settings (Cho et al., 2018; Kowalsky et al., 2019), with non-intrusive sensors, enabling the quantification of walking behaviors in a free-living environment over extended periods of time (Wang and Adamczyk, 2019). Among other walking parameters, stride speed can accurately be measured using a foot-worn IMU (Foxlin, 2005; Sabatini et al., 2005; Ojeda and Borenstein, 2007; Rebula et al., 2013). However, IMUs present several challenges that limit their current use in real-world, long-term monitoring of clinical populations. Many sensors of this type need to be recharged after daily use and are usually secured to the body using elastic straps, which can lead to compliance issues (e.g., sensors lose power and stop collecting data, sensors are not donned at the beginning of the day). Additionally, research-grade IMUs often require the use of specific software packages to configure/reconfigure the devices to collect and download data. Alternative types of mobile accelerometry exist, which solve both compliance and battery life issues, though at the cost of a decrease in resolution (decreased sampling rate, no measure of angular velocity). These accelerometer-only devices are lighter in weight, smaller in size, and can be embedded in a belt or shoe sole (Benocci et al., 2009; Shu et al., 2010; Motl et al., 2012), or worn on the leg for an extended period of time (Edwardson et al., 2017). With these devices, there is no direct approach to measure or calculate walking speed from the extracted signals. Some machine learning methods have been developed to estimate free-living walking speed from specific accelerometer-based sensors (Schimpl et al., 2011), or smartphones (Silsupadol et al., 2017; Shrestha and Won, 2018). However, the different models in these studies (Schimpl et al., 2011; Silsupadol et al., 2017; Shrestha and Won, 2018) were mostly tested and validated in a supervised environment, using short distance walks in hallways, treadmills, or a measuring wheel rather than in the real-world during unconstrained and unsupervised walking (Warmerdam et al., 2020). New validated models that derive speed using data from acceleration-only devices used in free-living environments remain an open research problem.

To help extend the wearable sensing paradigm to the clinical setting, we focus here on creating an efficient approach that combines fine-scale and low-resolution long-term measurements to estimate gait speed in the free-living environment. We use a low-power accelerometer secured on the thigh to address compliance and battery life issues; this sensor is used to make precise measures of stride frequency. Using a foot-worn IMU, high-resolution acceleration and angular rate measurements were used to calculate stride speed. Metrics from both systems were then leveraged in a custom-designed walking task to build a subject-specific data-driven walking model, mapping stride frequency to stride speed. To demonstrate the ecological validity of this model, walking data was collected in the free-living environment with both an IMU on the foot and the body-worn accelerometer. We define the accuracy of our model in estimating gait speed as the agreement between the model and IMU measurements, which have been used as a viable assessment of walking speed in an unsupervised real-world environment (Foxlin, 2005; Sabatini et al., 2005; Ojeda and Borenstein, 2007; Rebula et al., 2013). Once validated, this approach can then be used to perform monitoring on a week-long scale, with thorough quantification of the walking activity, to allow clinicians to gain a unique insight on the physical health of a subject in their natural environment.

## 2. Materials and Methods

### 2.1. Subjects

Ten subjects were recruited from a healthy population (Table 1). Leg length, defined as *l*_0_, was measured from the anterior superior iliac spine to the floor, without shoes. The experiments were conducted in the free-living environment of each subject as no specific setting was required. Each participant provided written consent on a form where the purpose and detailed experimental protocol was explained. This study was approved by the Institutional Review Board of the University of Michigan.

**Table 1:** Subject information.

	**Leg length l_0_ (m)**	**Height (cm)**	**Weight (kg)**	**Age (years)**	**Gender**
Subject 1	0.94	169	71	23	F
Subject 2	0.91	160	54	36	F
Subject 3	0.88	160	53	22	F
Subject 4	1.01	179	70	23	M
Subject 5	0.94	165	64	22	F
Subject 6	1.06	178	70	38	M
Subject 7	0.99	173	68	25	M
Subject 8	1.03	183	70	27	M
Subject 9	1.00	180	71	30	M
Subject 10	1.00	175	75	27	F

### 2.2. Instruments

#### 2.2.1. Free-Living Measurements

The activPAL^TM^ [PAL Technologies Ltd., Glasgow, UK] (AP) monitor is a low-power triaxial accelerometer-based bio-logging tag that can be placed on the thigh using an adhesive bandage tape. This lightweight (9g) waterproof sensor can be worn for 7 consecutive days without being removed or recharged. AP's compact size (23.5 × 43 × 5 mm) and shape make it an unobtrusive device; reducing the risk that a subject's behavior changes due to the consciousness of being studied (the Hawthorne effect) (Warmerdam et al., 2020). Proprietary algorithms enable classification of type and duration of different activity states (lying, sitting, standing, stepping, cycling, and driving). Summary metrics, such as the number of steps, sit-to-stand transitions, or hours in each activity state are also calculated and summarized from the data. The reliability of this sensor and associated algorithms has been validated in the literature (Edwardson et al., 2017).

**Figure 1:**
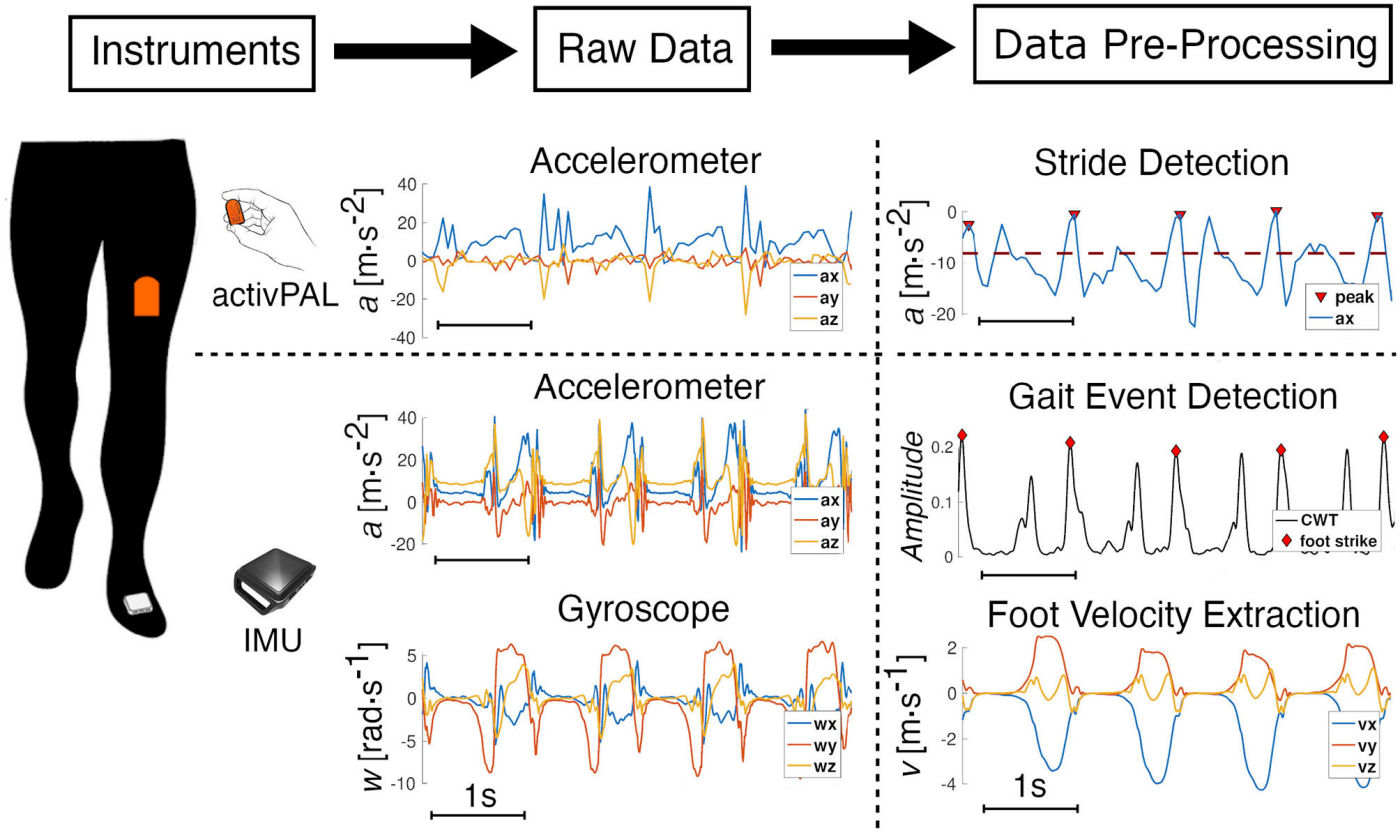
Data processing. Data extraction and pre-processing from the synchronized activPAL (AP) and the IMU. A representative window of four strides illustrates the pre-processing of the raw data from both sensors. The AP is worn on the thigh, and the accessible raw data is from the embedded three-axis accelerometer. The x-axis signal, which is from the sensor-axis that is aligned with the thigh and is positive in the inferior direction, is smoothed and a peak detection (threshold in red-dotted line) method enables the detection of each stride. For the IMU, raw data from the embedded three-axis accelerometer and gyroscope are processed using a continuous wavelet transform to detect gait events; foot velocity and position are then calculated using a ZUPT algorithm.

With a sampling rate of 20*Hz*, the acceleration measurements from the AP monitor can be used to accurately detect strides. The sensor is instructed to be placed on the anterior surface of the thigh with the positive x-axis pointing inferiorly, the positive y-axis pointing medially, and the positive z-axis to be pointing anteriorly out of the thigh. Using the classification of the different activity states, bouts labeled as “*Walking”* were extracted from the long-term measurements; short bouts (<10 s) were discarded. To extract gait events, we isolated the accelerometer signal from the x-axis of the sensor. The raw accelerometer signals from this axis was smoothed using a locally weighted scatterplot smoothing (LOWESS) method. This technique removed noise while keeping the main peaks of the signal. We applied a peak detection method to identify stride frequency for each smoothed, steady-state walking bout longer than 10 s in duration (Figure 1). Sections used for analysis were required to contain at least five strides. A section started when the first peak above a fixed threshold was detected. Using labeled data, we heuristically determined a threshold of −8*m* · *s*^−2^ for all participants, illustrated by a red dashed line in Figure 1. We defined a minimum time interval between peaks assuming a minimum stride time of 0.5 s, which corresponds to a very fast walking speed to make sure all strides were captured. We tracked the change in time between two consecutive peaks to identify incorrect peak detections or pauses in a walking bout. These identified outliers marked the end of a section. To account for the anthropometric differences between subjects, values of stride frequency were normalized by $\sqrt{g/l_{0}}$, where *l*_0_ is the subject's leg length and *g* is the value of standard gravity (Hof, 1996).

#### 2.2.2. High-Resolution Short-Term Measurements

IMUs offer a reliable alternative to other methods, such as optical or electromagnetic motion capture for capturing walking gait data (Cho et al., 2018). In this work we used the Opal sensor from APDM [Portland, OR, USA] which is a wearable IMU with a three-axis accelerometer (range ±200*g*), a three-axis gyroscope (range ±200*deg* · *s*^−1^), and a three-axis magnetometer (range ±8*Gauss*). The IMU was worn on the dorsum of the foot, on top of the shoe, using an elastic strap. The x-axis was positive in the distal direction and was aligned with the long axis of the foot, while the positive z-axis pointed superiorly out of the foot. The system was configured to record all signals at 128*Hz*, resulting in battery life of at least 8 h.

Continuous wavelet transforms (CWT) were used for gait event detection using inertial sensor signals from real-world gait (Khandelwal and Wickström, 2016). The continuous wavelet transforms were used to decompose the signal in the time-frequency domain to detect abrupt changes in a signal. In walking gait, foot strike events are one of the gait events that can be used to single out each gait cycle. They correspond to the impact of the foot on the ground, which creates a sudden variation in the foot acceleration. CWT can then be used on the raw acceleration signal to identify these events. Using custom code written in MATLAB (MathWorks, Inc., Natick, MA, USA), high-frequency content (>40 Hz) was kept from the CWT of the accelerations along the three axes, to ensure the analysis was insensitive to sensor orientation. The norm of each transform was summed, and using a peak detection method, we were able to identify foot strike events (Figure 1).

After identifying foot strikes, we utilized the zero-velocity update (ZUPT) algorithm to calculate stride lengths. ZUPT is a well-documented technique commonly used in pedestrian navigation systems (Foxlin, 2005; Ojeda and Borenstein, 2007) and provides highly accurate estimates of foot position and stride parameters (Rebula et al., 2013; Potter et al., 2019). IMU estimates of mean stride length and duration have been found to be within 1% of motion capture for walking. The ZUPT algorithm (Foxlin, 2005; Ojeda and Borenstein, 2007; Rebula et al., 2013; Potter et al., 2019) uses the assumption that the velocity of the foot on the ground reaches zero or close to zero and uses these points of known zero velocity to correct the drift in the estimates of foot velocity. The implementation we used followed the formulation that is presented and evaluated in the work of Rebula et al. (2013) and Potter et al. (2019) (which also uses the same hardware). With this method, stride speed and stride length, among other gait parameters, can be accurately calculated. Stride speed was normalized by $\sqrt{g\cdot l_{0}}$, and stride length by *l*_0_ (Hof, 1996).

### 2.3. Subject-Specific Model Derivation

Walking is a cyclic motion of the lower limbs, which can be described by parameters from the time and frequency domain. To minimize the energy consumption of the body, individuals naturally adapt some of these parameters to their walking speed. Particularly, data collected from individuals walking at different speeds have shown that, for each individual, there is a preferred stride length *d* for a chosen stride speed *v* (Grieve, 1968):

(1) $$\begin{array}{r} d=a\cdot v^{b} \end{array}$$

where *a* and *b* are the parameters of the model, specific to each individual. Later, a simple walking model predicted the same relationship for a minimal metabolic rate in human walking (Kuo, 2001). Since stride frequency, *f*, can be expressed as the ratio *v*/*d*, Equation (1) can be used to calculate stride speed as a function of stride frequency:

(2) $$\begin{array}{r} v=\exp\frac{\ln\left(a\cdot f\right)}{1-b} \end{array}$$

In the current study, the parameters *a* and *b* were identified using data from a foot-worn IMU during a “metronome walk” conducted by each subject. In the metronome walk, subjects walked with a wide range of stride frequencies over a flat terrain. The location of the test was different for each participant. Overall, the flat terrains chosen were between 100 and 300 m over the duration of the test. Step frequencies were imposed by a metronome set at 45, 60, 75, 90, 105, 120, 135, and 150 bpm. Participants traveled back and forth on the terrain at each step frequency, allowing the sensors to collect a minimum of 10 strides per metronome frequency. The overall time to execute the entire protocol was ~15 min for every participant. Subjects were asked to synchronize their steps as best as they could to the beats of the metronome. This protocol was designed to capture a full range of walking frequencies for each individual while walking in an unconstrained environment.

**Figure 2:**
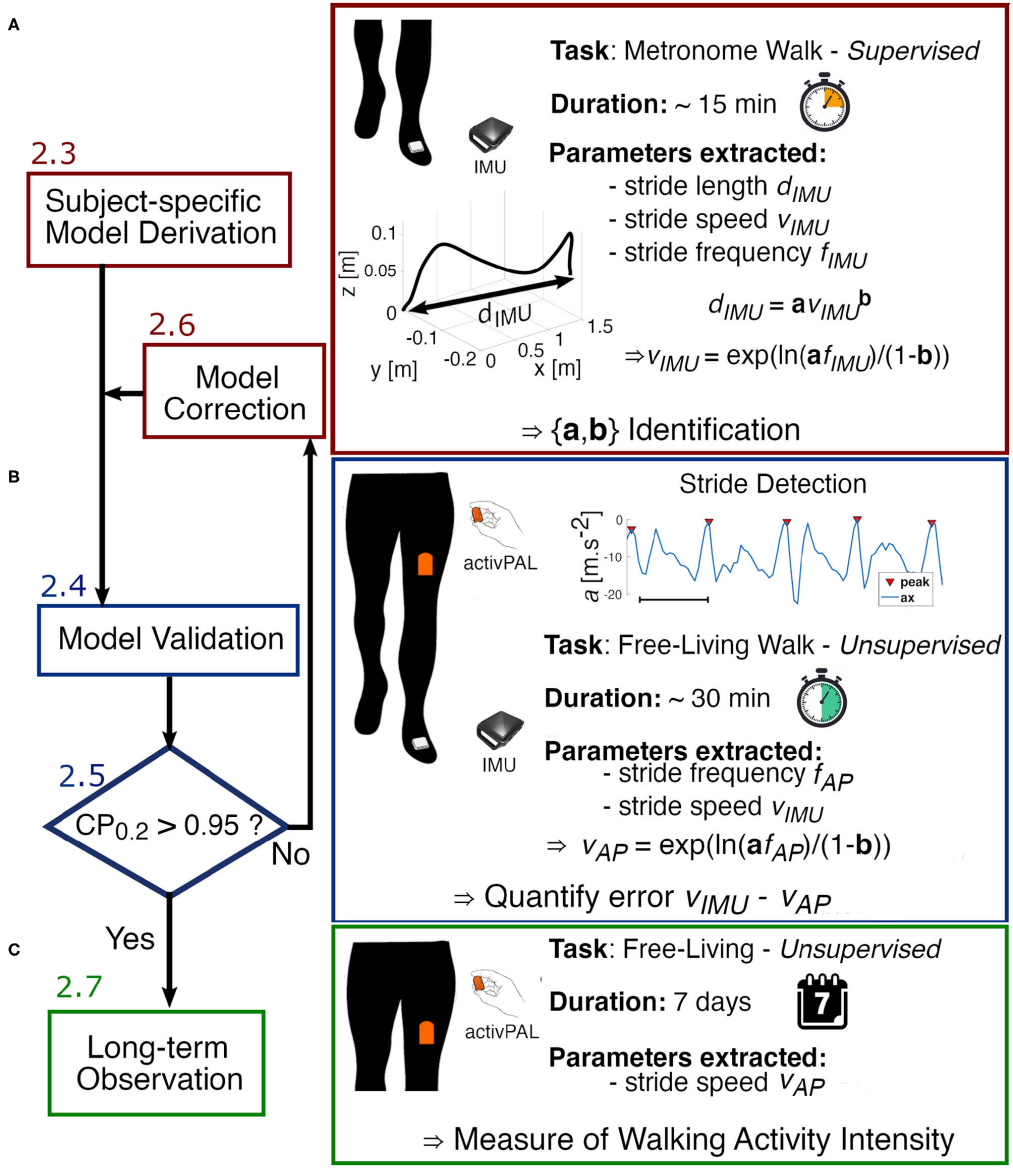
Experimental flow chart. **(A)** We start by deriving the parameters *a* and *b* of the power model using data collected from the IMU worn on the foot during the designed Metronome Walk. **(B)** To verify the accuracy of the model and validate the identified parameters, each subject performs a long unsupervised walk in the real-world wearing both the activPAL (AP) on the thigh and the IMU on the foot. The error between the estimated speed from the AP and the speed extracted from the IMU is then analyzed. If the error is not clinically acceptable (Coverage probability with boundaries ±0.2*m* · *s*^−1^ *CP*_0.2_ > 0.95), the model is adjusted by finding new parameters *a*′ and *b*′. **(C)** Once the model is validated, we can proceed to a longer observation with the AP only.

We define stride speed and stride length extracted from the IMU as *v*_*IMU*_ and *d*_*IMU*_, respectively. These two variables were extracted from each stride of the different walking bouts (defined by stride frequency) using the method described in 2.2.2. to identify *a* and *b*. Since subjects take a couple of steps to adapt to each stride frequency and tend to slow down toward the end of each walking bout, the transition steps from the beginning and the end of each walking bout were removed from the analysis. Through this designed protocol, we identified the parameters *a* and *b* unique to each subject using MATLAB's curve fitting toolbox. This toolbox uses least squares analysis, in our case non-linear least squares, to fit the data with the power model. We used the default fit options for our analysis (Figure 2A).

### 2.4. Model Evaluation

The model parameters *a* and *b* were determined using measures of stride frequency and stride speed from a prescribed and supervised walk in an unconstrained environment. Thus, there was no certainty that the identified parameters remain the same if walking data was collected in the free-living (unsupervised) environment. Therefore, we designed a protocol to verify that our model can be used in the real-world (unsupervised and unconstrained environment).

Each subject performed a long unsupervised walk at their self-selected pace, with no specific time or environment (inside or outside) constraints, while wearing both an IMU on the foot and the AP on the thigh. Each participant was shown how to place the sensors on themselves through a demonstration. Sensor locations were then verified on each user by examining the raw data. Note that orientation of the sensors did not influence the analysis. The participants were advised to complete a minimum of 30 min of walking in total but were free to use the time as they saw fit. To analyze the same walking bouts using both sensors, the IMU data were downsampled and synchronized with the data from the AP. Then, we identified steady-state walking bouts from which we extracted stride frequency *f*_*AP*_ from the thigh-worn accelerometer signal using the methodology introduced in 2.2.1. We used the subject-specific models (created as described in 2.3) to map each value of stride frequency to its corresponding stride speed *v*_*AP*_. To quantify the error associated with these estimates of stride speed, a ground truth measure of speed was established by leveraging the accuracy of the IMU using the method described in 2.2.2. Therefore, with this protocol, we evaluated the performance of our model in an uncontrolled environment over an extended period of time during which natural walking behaviors can be observed (Figure 2B).

### 2.5. Statistical Analysis

#### 2.5.1. Goodness of Fit of the Subject-Specific Model

We evaluated the subject-specific models that were built for each subject to quantify the confidence in the identified parameters *a* and *b*. The parameters used to evaluate the goodness of fit of the power regression are R-squared (coefficient of determination), the confidence intervals around the identified parameters, and the root mean square error (RMSE). R-squared is a statistic that quantifies how well our models explain the variation of the data. The confidence intervals of *a* and *b* quantify the confidence in the fitted coefficients and provide significant insight into whether or not more data are needed to build the model. The RMSE, also known as the standard error of regression, is an assessment of how spread out the data are around the regression line.

#### 2.5.2. Analysis of the Residuals

Data from the unsupervised long walk were used to evaluate the accuracy of the subject-specific models for predicting walking speed. We utilized multiple statistical methods to allow for direct comparison with previous and future related work. We used the Bland-Altman analysis to assess the concurrence between the estimated speed from our model and the ground truth walking speed calculated from the foot-worn IMU data. The Bland-Altman analysis is a data visualization tool based on the study of the mean difference and the definition of limits of agreements within which 95% of the data falls (Giavarina, 2015). The distribution of the points around the mean difference and their distance to this value are visual and numerical indicators of the agreement between the estimated (from AP) and the true values (from the foot-mounted IMU) of walking speed. Besides the visualization of the error, the randomness of the points can reveal whether the model chosen (e.g., the power model) is appropriate. By visualizing the Q-Q plot for the estimated and true speed, we confirmed normality of the distributions was a reasonably good approximation.

To complement this analysis, we calculated two other statistical metrics to assess the validity of the created model. The coverage probability (*CP*_κ_) represents the probability *Pr* that the residuals will be within a defined boundary κ (Lin et al., 2002). In our study, it can be translated by the following equation:

(3) $$\begin{array}{r} CP_{\kappa }=Pr\left(|v_{IMU}-v_{AP}|<\kappa \right) \end{array}$$

Three different boundaries were evaluated at 0.1, 0.2, and 0.3*m* · *s*^−1^. To be able to confidently use the model created in a clinical setting, the error observed between the true and estimated stride speed should be within the minimal clinically important difference. For gait speed, it has been established that the minimal clinically important difference is between 0.1 and 0.2*m* · *s*^−1^ (Bohannon and Glenney, 2014). Thus, we considered a subject's model valid if 95% of the residuals were within the ±0.2*m* · *s*^−1^. We also calculated the concordance correlation coefficient (CCC). This metric has been created to measure the variation of measures (*X, Y*) from the *X* = *Y* line (Lin, 1989). It represents a more appropriate way to evaluate the correlation between two measurements than the traditional Pearson correlation coefficient, as it captures any location or scale shift in measurements. It can take any value between 0 and 1, with 1 being a perfect concordance between the measurements.

### 2.6. Model Correction

If the analysis of the residuals revealed a clinically significant difference for a subject, with <95% of the residuals within ±0.2*m* · *s*^−1^, corresponding to *CP*_0.2_ > 0.95, we did an exploratory analysis of the metronome walk data to determine the source of discrepancies between true and estimated speed. We started with an observation of the statistical analyses to localize if a specific range of stride speed was being estimated poorly. This enabled us to find which data in the metronome walk was not representative of the natural behavior of the subject. By combining these observations with the knowledge of the effects in the variation of the parameters of the power model, we designed an iterative algorithm to obtain better estimates of the true parameters *a* and *b*. This algorithm started with an initial guess of the parameters based on the observation made on the primary data collected and terminated when the analysis of the residuals led to a valid model as defined in section 2.5.2 (Figure 2A).

Then, we investigated the effects of variations in the parameters *a* and *b* to visualize the underlying biomechanics behind the power model. By fixing a parameter and changing the other, we can isolate the influence of each parameter on the shape of the power curve.

### 2.7. Week-Long Quantification of the Walking Activity

Once the models were validated, we collected data for 1 week from the AP worn by one participant from this study (Figure 2C). We identified steady state walking bouts from the raw accelerometer signal as explained in section 2.2.1. Then, we used the model that we built and validated to estimate stride speed from each walking bout. We investigated walking speed across different time scales within a week to explore how the data could be used in future work.

## 3. Results

### 3.1. Mapping From Stride Frequency to Stride Speed

**Figure 3:**
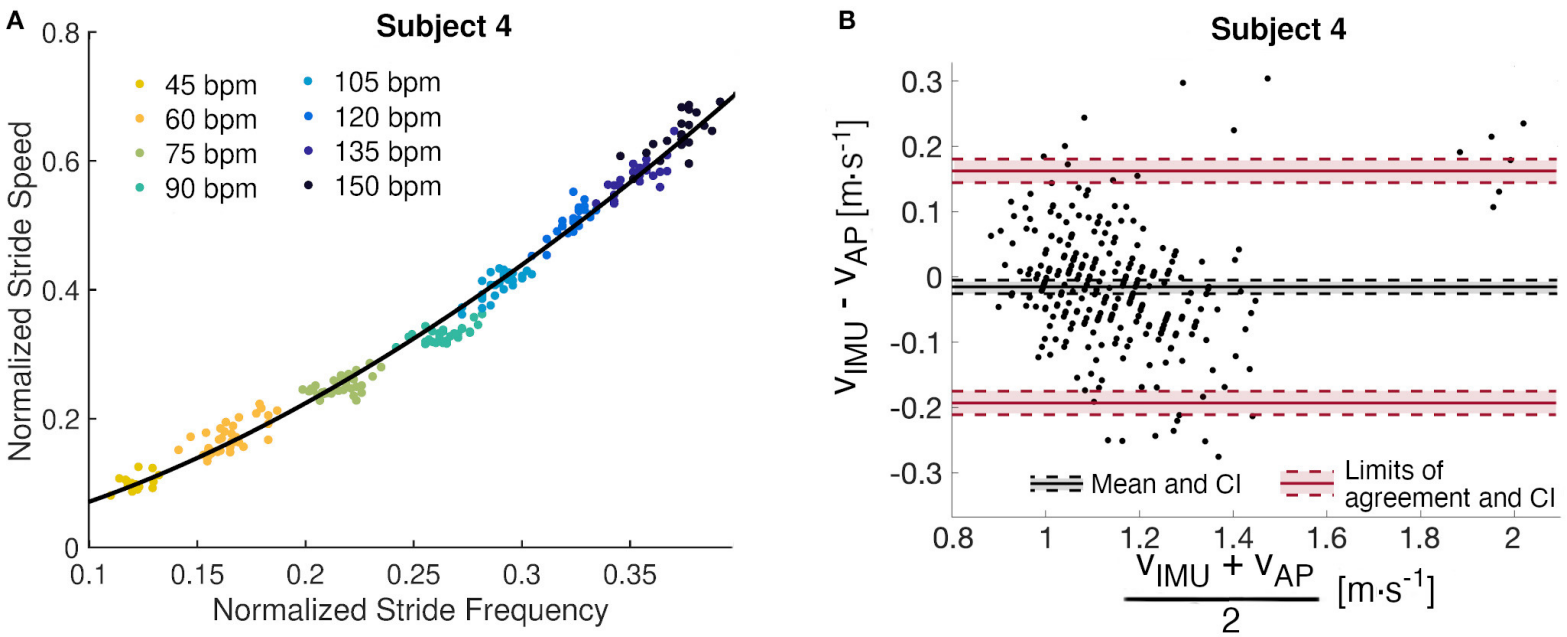
Subject 4 analysis. **(A)** Subject-specific data-driven map from stride frequency to stride speed built from the metronome walk. Each cloud of points corresponds to an imposed stride frequency. **(B)** Bland Altman analysis of the residuals with mean difference, limits of agreement, and their confidence intervals (CI).

Data from the subjects' metronome walk were used to identify parameters for the power regression model (*a* and *b*). The subject-specific models account for at least 97.8% of the variance, and the maximum RMSE is 0.013 *m* · *s*^−1^, which indicates a good fit for all models (Table 2). We observed maximum confidence interval widths of 0.081 and 0.034 for *a* and *b*, respectively (Table 2). The parameters *a* and *b* vary from 1.961 to 2.295 and 0.292 to 0.397, respectively (Table 2). The fitted line for a representative subject illustrates the utility of the designed walking task (Figure 3A), which resulted in a wide range of walking frequencies and speeds for model identification, where distinct clouds of points correspond to each prescribed stride frequency and the corresponding range of stride speeds.

**Table 2:** Goodness of fit.

	**a**	**b**	**R-squared**	**CI a**	**CI b**	**RMSE**
Subject 1	2.009	0.292	0.992	(1.991, 2.027)	(0.283, 0.300)	0.008
Subject 2	2.050	0.335	0.987	(2.017, 2.084)	(0.319, 0.352)	0.009
Subject 3	1.997	0.314	0.981	(1.948, 2.006)	(0.301, 0.328)	0.011
Subject 4	2.028	0.397	0.986	(2.005, 2.051)	(0.386, 0.408)	0.009
Subject 5	2.109	0.372	0.984	(2.068, 2.149)	(0.356, 0.389)	0.011
Subject 6	2.014	0.375	0.991	(1.998, 2.03)	(0.367, 0.382)	0.009
Subject 7	2.002	0.305	0.992	(1.98, 2.025)	(0.295, 0.316)	0.008
Subject 8	1.961	0.324	0.991	(1.938, 1.985)	(0.313, 0.335)	0.009
Subject 9	2.295	0.392	0.978	(2.259, 2.331)	(0.376, 0.407)	0.013
Subject 10	2.173	0.384	0.986	(2.137, 2.208)	(0.368, 0.400)	0.010

*Analysis of the goodness of fit of the power model built during the Metronome Walk for each subject. CI, Confidence interval; RMSE, Root mean square error*.

### 3.2. Quantification of the Walking Speed Estimate Error

**Figure 4:**
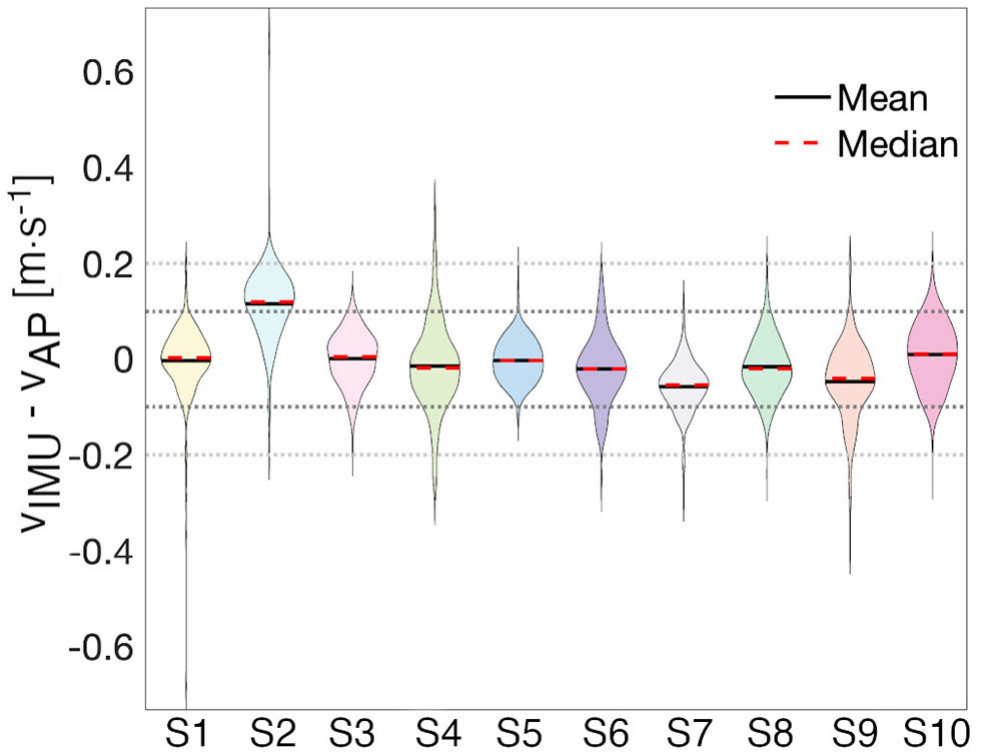
Distribution of residuals. The violin plot enables the visualization of the distribution of the error for each subject with ±0.1*m* · *s*^−1^ (black-dotted line) and ±0.2*m* · *s*^−1^ (gray-dotted line) bands. Each shaded area represents the shape of the distribution of residuals for a particular subject.

The Bland-Altman analysis indicates that all subjects, except Subject 2 and Subject 9, have limits of agreement within ±0.2*m* · *s*^−1^ (Table 3). Subject 2 also presents the highest mean difference between estimated and true walking speed, followed by Subject 7 and Subject 9 (Table 2 and Figure 4). The violin plot (Figure 4) shows that majority of residuals fall into the ±0.1*m* · *s*^−1^ range, except for Subject 2, where we observe that the true speed is clearly underestimated by the model. The other subjects have small mean differences between −0.058 and 0.008*m* · *s*^−1^. The Bland-Altman plot for a representative subject of this study, Subject 4, shows a concentration of the points between 0.8 and 1.4*m* · *s*^−1^ (Figure 3B).

**Table 3:** Bland-Altman analysis.

	**Sample size**	****μ(*v*_*IMU*_ − *v*_*AP*_)****	****σ(*v*_*IMU*_ − *v*_*AP*_)****	**Limits of agreement**	
				**Inferior**	**Superior**
Subject 1	245	−0.013	0.078	−0.157	0.148
Subject 2	248	0.116	0.084	−0.048	0.280
Subject 3	465	0.000	0.054	−0.107	0.107
Subject 4	294	−0.015	0.091	−0.193	0.162
Subject 5	449	−0.004	0.044	−0.091	0.083
Subject 6	144	−0.021	0.071	−0.159	0.117
Subject 7	530	−0.058	0.053	−0.162	0.045
Subject 8	482	−0.017	0.061	−0.137	0.104
Subject 9	534	−0.048	0.079	−0.203	0.108
Subject 10	478	0.009	0.067	−0.123	0.140

*Bland & Altman plot statistics for all subjects. μ and σ correspond to the mean and standard deviation, respectively*.

To quantify the agreement of the estimated speed from the AP and the speed extracted from the IMU, we calculate CPs for each subject for three different clinically meaningful boundaries and computed the CCC (Table 4). All subjects' data, except Subject 2's, contain at least 70% of the error within ±0.1*m* · *s*^−1^ and at least 97% within ±0.2*m* · *s*^−1^. An analysis of all subjects suggests that 97% of the residuals are within ±0.2*m* · *s*^−1^. The CCC values range from 0.65 for Subject 2 to 0.96 for Subject 6, with an average of 0.80.

**Table 4:** Measures of agreement.

	**CP_**0.1**_**	**CP_**0.2**_**	**CP_**0.3**_**	**CCC**	**CCC CI inf**	**CCC CI sup**
Subject 1	0.80	0.99	1.00	0.82	0.78	0.86
Subject 2	0.33	0.78	0.98	0.65	0.60	0.70
Subject 3	0.93	1.00	1.00	0.90	0.88	0.92
Subject 4	0.72	0.97	1.00	0.86	0.83	0.89
Subject 5	0.97	1.00	1.00	0.91	0.89	0.92
Subject 6	0.82	0.99	1.00	0.96	0.95	0.97
Subject 7	0.78	1.00	1.00	0.62	0.58	0.66
Subject 8	0.88	1.00	1.00	0.78	0.74	0.81
Subject 9	0.71	0.97	1.00	0.66	0.61	0.70
Subject 10	0.86	1.00	1.00	0.84	0.82	0.86
Average	0.78	0.97	1.00	0.80	0.77	0.83

*Coverage probabilities (CP) for the boundaries 0.1, 0.2, and 0.30.013m · s^−1^ and concordance correlation coefficient (CCC) with its confidence interval (CI)*.

**Figure 5:**
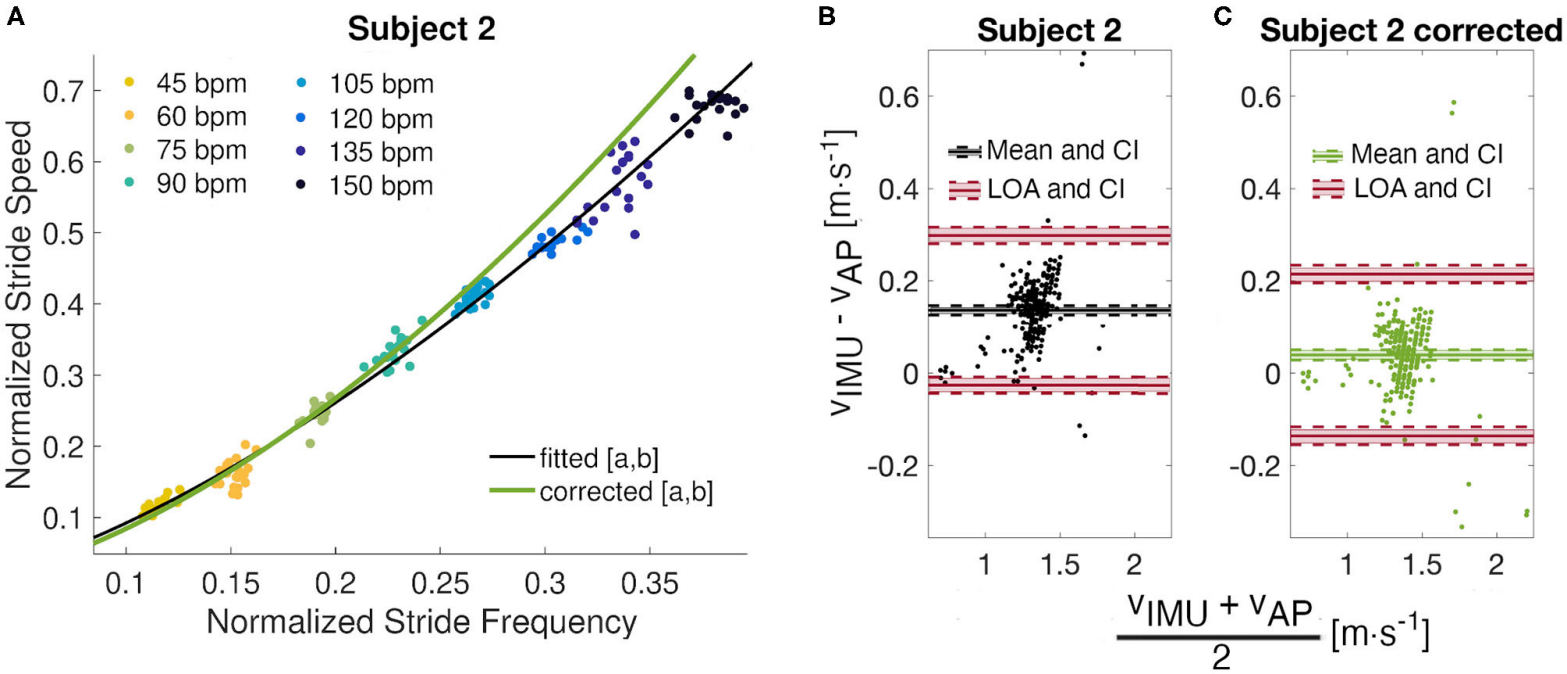
Model correction. **(A)** Observation of the metronome walk fitted curve (*a* = 2.050 and *b* = 0.335) against the corrected power model (*a*′ = 2.266 and *b*′ = 0.399) for Subject 2. **(B)** Bland Altman analyses for the original and **(C)** corrected models with mean differences, limits of agreements, and their confidence intervals (CI).

Results from Subject 2 show a large difference (*CP*_0.2_ > 0.95) between estimated and true walking speeds. Indeed, only 78%, instead of 95%, of the residuals were within the ±0.2*m* · *s*^−1^ boundary. The Bland-Altman plot for this individual (Figure 5B) illustrates a small cloud of points, corresponding to low values of stride speeds, around zero. From this observation, we identified different parameters *a*′ = 2.266 and *b*′ = 0.399 with respective confidence intervals (2.161, 2.370) and (0.369, 0.431) by fitting a power model only to the four slowest stride frequencies (Figure 5A). The R-squared for this fit is 0.961 and the RMSE is 0.008. A new analysis of the residuals revealed better performance of the updated model, with a mean error of only 0.039*m* · *s*^−1^ and limits of agreements of 0.214 and −0.136*m* · *s*^−1^ (Figure 5C). The new CP values indicate 69% of the error within ±0.1*m* · *s*^−1^ and 96% within ±0.2*m* · *s*^−1^. The CCC of the corrected model is 0.85 compared to 0.65 for the fitted model.

### 3.3. A Week in the Life of Subject 1

**Figure 6:**
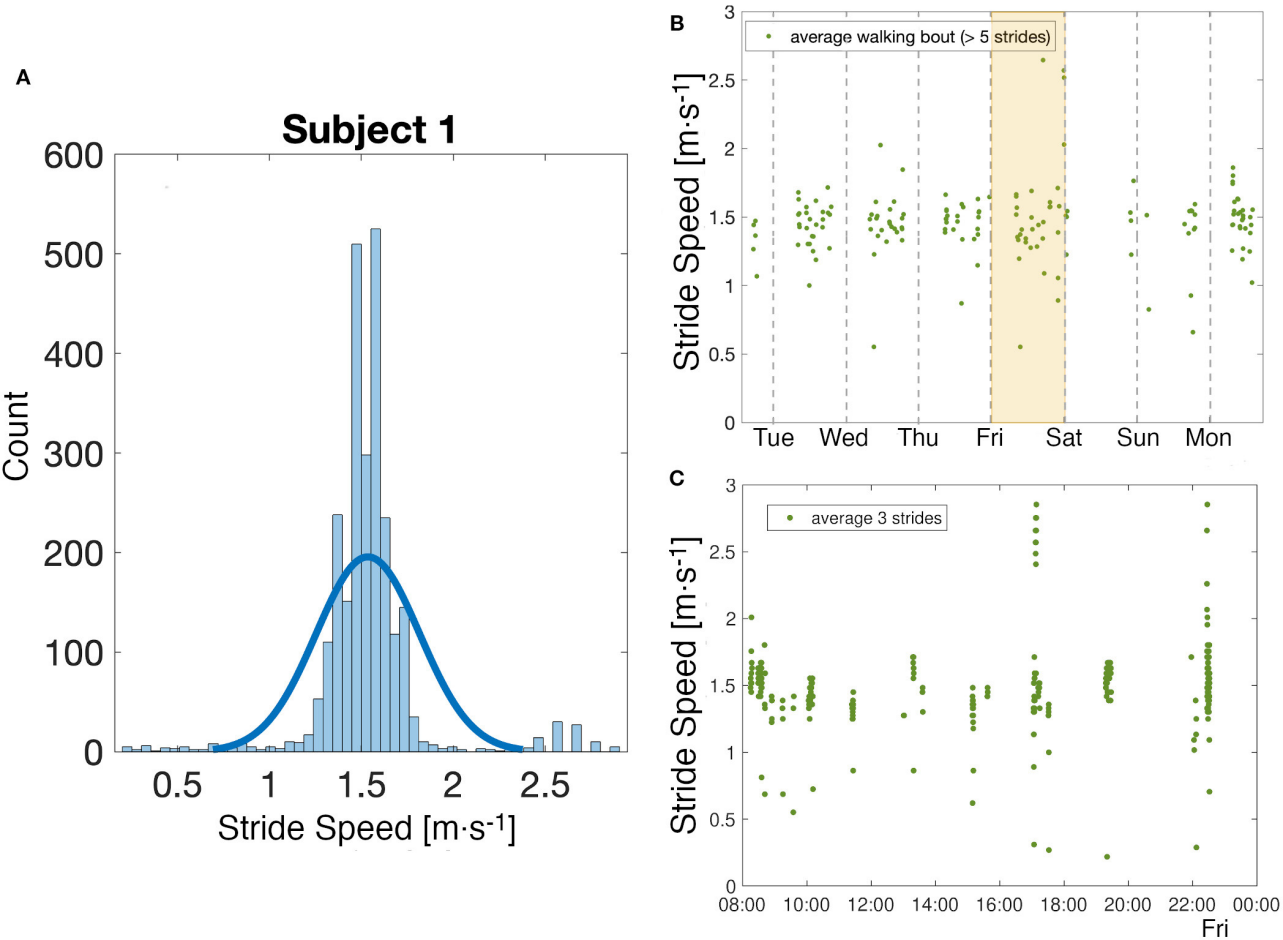
Week analysis. **(A)** Analysis of the distribution of all stride speed values collected over a week for one subject. **(B)** Observation of the evolution of walking speed in a week. Each point corresponds to the average of the values of stride speed in a quasi-steady-state walking bout as defined in Methods. **(C)** A closer look at the values of stride speed on a particular day, shaded in yellow in the figure above. Each point corresponds to the average of three strides.

During the week long experiment, 168 h of data were collected from subject 1. The histogram of the average steady state speed indicates a strong preference to walk at 1.47 and 1.58*m* · *s*^−1^ (Figure 6A). During that time, 167 bouts of walking (as defined in Methods) were identified with an average of 16 strides per bout. Overall, the subject had an average steady state speed of 1.53*m* · *s*^−1^. There were not large daily differences in the data, with the slowest average day of 1.36*m* · *s*^−1^ and the fastest of 1.50*m* · *s*^−1^ (Figure 6B). Figure 6C illustrates the variability in stride speed over the course of a day; 85% of the data is contained within 1.00 and 1.80*m* · *s*^−1^, with a maximum speed recorded around 5 p.m. at 2.85*m* · *s*^−1^ and a minimum speed recorded around 7:20 p.m. at 0.22*m* · *s*^−1^.

## 4. Discussion

Walking is an important activity of daily life for many individuals, and the ability to monitor and assess gait in a free-living environment is key to an improved understanding of an individual's physical health. Here, we utilized fine-scale measurements from a prescribed walking task to build subject-specific walking models that accurately estimate gait speed from low-resolution long-term accelerometers, in an unconstrained environment. Finally, a representative subject wore the bio-logging tag for a week to demonstrate the viability of this approach for the extraction of clinically relevant parameters in the real-world.

Data to generate the subject-specific model parameters were collected using a metronome to create a large range of stride speeds and frequencies without the constraints that result from walking over a range of fixed speeds on a treadmill (Bertram and Ruina, 2001). The identified parameters (*a* and *b*) for the power model are similar in magnitude with the first study (to our knowledge) which used this model (Grieve and Gear, 1966). The goodness of fit measures presented in Table 2 further demonstrate that the power model captures the relationship between stride speed and frequency well. Figure 3B illustrates a random and equal distribution of points around a zero mean difference in the Bland Altman plots, which can be used to verify the modeled relationship between stride frequency and speed.

**Figure 7:**
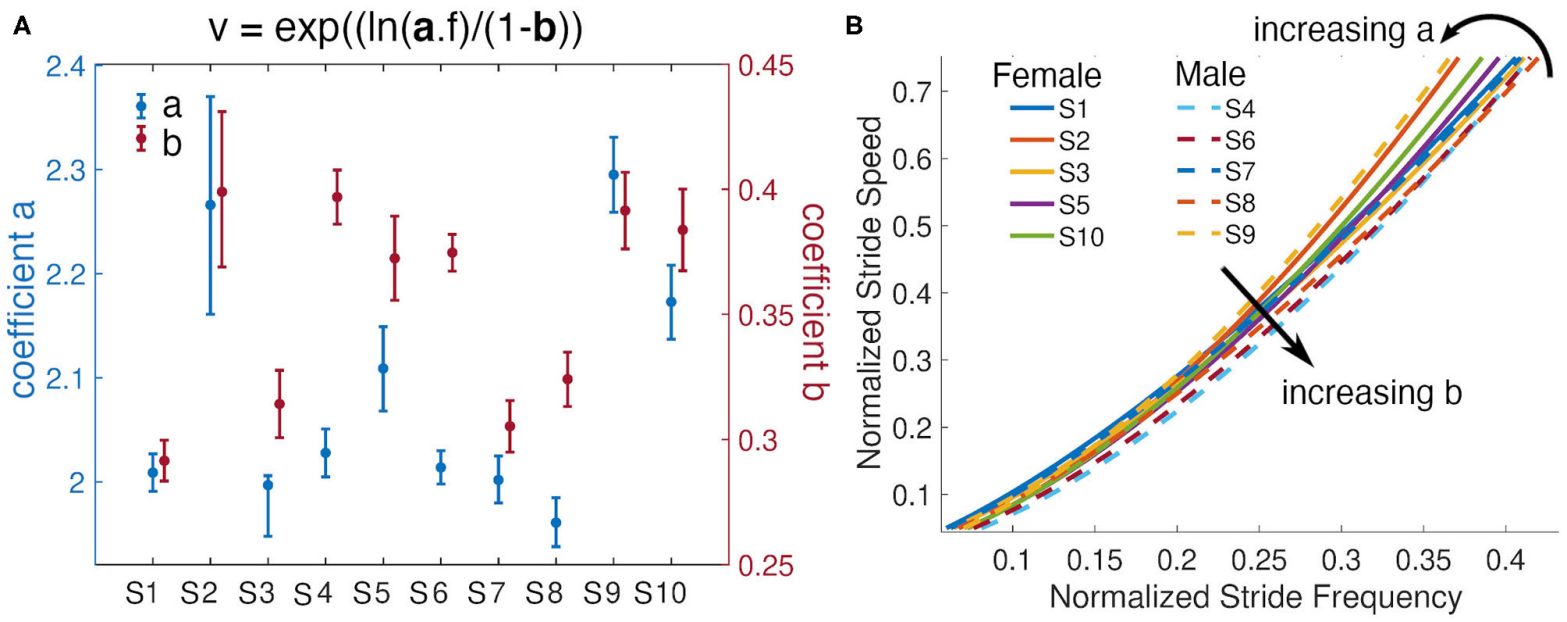
Subjects' models and their parameters. **(A)** Parameters *a* and *b* from each subjects power model and their 95% confidence intervals. **(B)** Fitted lines for all subjects and visualization of the effects of variations of the parameters *a* and *b*. Females are represented with solid lines and males with dashed lines.

Analysis of the subject-specific models indicate that individuals with similar anthropometric parameters do not always have the same relationship between stride speed and frequency. Subjects 4 and 9 have almost equal leg lengths and heights, but their identified parameters *a* differ by 0.267. Subject 9's fit resulted in the largest *a* value, while Subject 4's fit has a fairly small *a* but the second largest *b* value (Figure 7A). To achieve a given speed, Subject 9 used longer strides at a lower frequency, while Subject 4 employed shorter steps at a higher frequency (Figure 7B). Environmental or contextual differences, like the type of footwear (Wang and Adamczyk, 2019), might also influence the parameters of the model and warrant further investigation. For example, Wang and Adamczyk have shown that gait parameters can change between walking with sandals and athletic shoes. All participants wore athletic shoes for this study. Additionally, the analysis of Subject 2's statistics indicated that the walking speeds at the higher step frequencies were slower than during free walking. All subjects were given limited time just before the task to familiarize themselves with the metronome walk before data was recorded. In the future, more time to learn and practice the metronome walk could improve the data utilized for the model identification.

**Figure 8:**
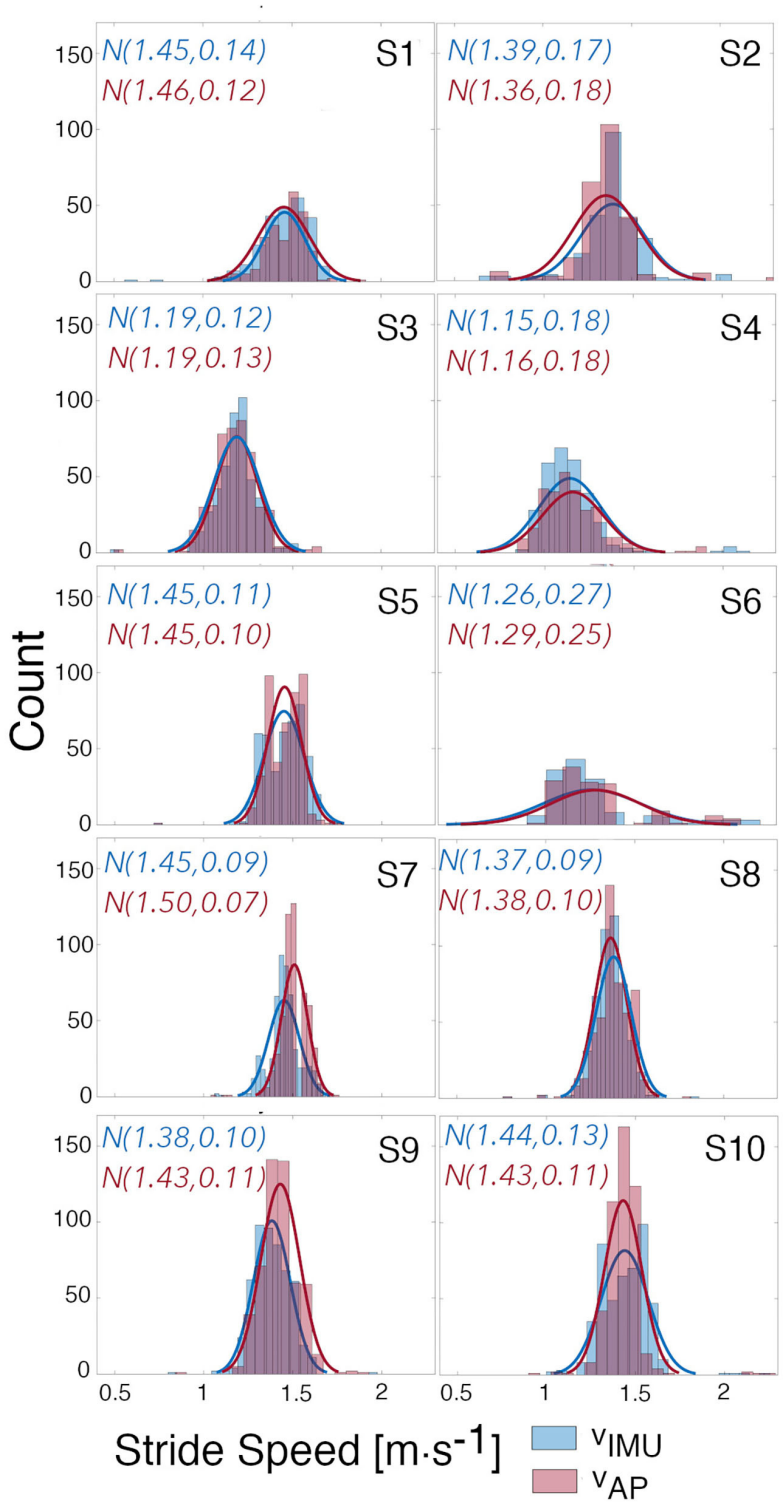
Distributions of estimated and true speed. Distributions of the estimated speeds from the AP using the built models, and of the true speeds calculated from the IMU. Gaussian curves were fitted to each distribution and mean and standard deviation for each curve are given.

To evaluate our approach, estimates of stride speed calculated from the foot-worn IMU were compared to the model-based estimates during an unsupervised long walk in the real-world for each subject (Figure 8); for Subject 2 the corrected model was used. Qualitatively, the speed distribution for the two estimates are in good agreement for all subjects. Quantitatively, more than 95% of the error between the estimated speed from our model and the speed extracted from the foot-worn IMU were within ±0.2*m* · *s*^−1^, with the estimated mean walking speed comparing well to the true value for the majority of subjects. The CCC values can be visualized by looking at the overlapping regions of the distributions of *v*_*IMU*_ and *v*_*AP*_. Subject 6 presents almost a perfect overlap with a corresponding CCC value of 0.95 whereas there is a slight shift in Subject 7's distributions who presents a CCC value of 0.58. The study from Schimpl et al. (Schimpl et al., 2011) compared different methods to extract stride speed from a single waist-worn accelerometer in the free-living environment. The method that gave the best results when tested during outdoor walking with self-selected speed used a support vector regression algorithm. The values of CP and CCC reported by this study when using this method are comparable with the value obtained in our study. The Bland Altman analysis of the results from the thigh-worn accelerometer also have smaller bias and limits of agreements for estimated walking speed than results in the literature that used smartphone data collected at different locations (e.g., body, bag, belt, hand, and pocket) to estimate speed (Silsupadol et al., 2017). This points to the benefit of data collection from a fixed point on the lower body.

Extending this work to the clinical environment will require data collection over multiple days in the real-world environment. The wearable sensor used in this work is capable of capturing 7 days of continuous motion data, and the attachment on the thigh enables unobtrusive data acquisition in the free-living environment. Using this sensor platform we were able to collect data for a week from a single representative subject of this study. The week-long assessment demonstrates the potential of this approach to gain a unique insight into movement and activity at different temporal resolutions; we expect similar results when we extend our approach to assess a larger subject population. The capacity to extract average stride speeds from 167 bouts of walking (duration >10 s and strides >5) enables estimation of an individuals preferred walking speed in their daily environment. In the future, we expect that the extraction of walking speed, in combination with other parameters, over an extended time period could allow clinicians to get a more objective and complete picture of the walking activity of individuals in their free-living environment, compared to a traditional in-laboratory walking test. Fine grain observations of daily trends could be used to build more tailored prescription related to physical activity.

A potential shortcoming in this study is the use of an IMU-based estimate of stride speed to derive and verify the speed estimates presented in this work. IMU-based estimates are known to contain small measurement errors on the order of 1 − 5% depending on gait speed (Foxlin, 2005; Ojeda and Borenstein, 2007; Rebula et al., 2013; Potter et al., 2019). Multi-camera motion capture and force plates are tools that have been proved to be highly accurate to estimate gait parameters (Chiari et al., 2005; Pfister et al., 2014). However, these measuring instruments cannot be used outside the laboratory and constrain data collection to a limited capture volume. Therefore, the ZUPT approach is one of the most viable method to accurately and unobtrusively assess the ecological validity of walking gait measures in the real-world. To our knowledge, the potential errors of the ZUPT algorithm have not been assessed in the free-living environment, since most validation methods are bound to the laboratory setting. However, the method has been validated during uncontrolled hallway walking with a large number of strides collected (Rebula et al., 2013). The conditions of this study are close to the walking conditions in which subjects performed the model validation. This makes us confident regarding our choice to use the ZUPT algorithm to obtain ground truth stride speed in this study. Another limitation to consider is the sample size and the population recruited in this work. Our experiment involved young healthy subjects. However, different studies have shown the impact of weight (Błaszczyk et al., 2011; Jegede et al., 2017) or age (Samson et al., 2001; Aboutorabi et al., 2016) on gait parameters. Additional work is needed to evaluate whether the power model can still be used with different study populations. In addition, the frequencies used during the metronome walk might also need to be adapted to participant or patient ability. This approach could also be enhanced by including parameters to inform how behavior of an individual extracted from a bio-logging sensor is interpreted. For example, being able to add a layer of physiological and contextual data on to the current analysis would allow a more objective evaluation of a patients' level of physical activity (Chen et al., 2012). These types of additions will further enable clinicians to design and monitor the compliance to tailored treatments to improve patient care and outcomes.

## Data Availability Statement

The raw data supporting the conclusions of this article will be made available by the authors, without undue reservation.

## Ethics Statement

The studies involving human participants were reviewed and approved by Institutional Review Board of the University of Michigan. The patients/participants provided their written informed consent to participate in this study.

## Author Contributions

LB: design, conception of the study, data collection, algorithms design, data analysis, data interpretation, manuscript drafting, and critical revisions. SC and KS: design, conception of the study, algorithms design, data interpretation, and critical revisions on the manuscript. KB: design, conception of the study, data interpretation, and critical revisions on the manuscript. MN and EJ: design of the study and secure funding. All authors agreed to be accountable for all aspects of the work.

## Conflict of Interest

The authors declare that the research was conducted in the absence of any commercial or financial relationships that could be construed as a potential conflict of interest.
